# The Role and Impact of Non-driver Gene Mutations in Myelofibrosis

**DOI:** 10.1007/s11899-026-00773-6

**Published:** 2026-02-10

**Authors:** Valentina Boldrini, Paola Guglielmelli, Alessandro M. Vannucchi

**Affiliations:** Department of Experimental and Clinical Medicine, Center for Research and Innovation of Myeloproliferative Neoplasms, Azienda Ospedaliera Universitaria Careggi, CRIMM, University of Florence, Largo Giovanni Brambilla 3, Florence, 50134 Italy

**Keywords:** Myelofibrosis, Mutations, Architecture, Hematopoietic stem cells, Mouse model

## Abstract

**Purpose of the Review:**

Myelofibrosis (MF) is a myeloproliferative neoplasm (MPN) characterized by splenomegaly, constitutional symptoms, bone marrow fibrosis and potential progression to a blast phase. This review provides a comprehensive overview of the current molecular landscape of MF beyond canonical driver mutations (*JAK2*,* MPL or CALR)*, emphasizing insights gained from murine models that served as valuable tools for understanding disease mechanisms.

**Recent Findings:**

High-throughput next-generation sequencing (NGS) has markedly enhanced our understanding of the molecular basis of MF, identifying numerous mutations beyond the canonical driver genes *JAK2*,* MPL*, and *CALR*, which are present in about 80% of patients. Additional mutations affect genes involved in DNA methylation (*TET2*,* DNMT3A*,* IDH1*,* IDH2*), histone modification (*ASXL1*,* EZH2*), mRNA splicing (*SF3B1*,* SRSF2*,* U2AF1*,* ZRSR2*), signaling pathways (*CBL*,* NRAS*,* KRAS*), and key transcription factors (*RUNX1*,* NFE2*,* TP53*). The presence and combination of these alterations influence clinical presentation, prognosis, and therapeutic response.

**Summary:**

This review offers an updated synthesis of the evolving molecular landscape of MF, highlighting how the intricate interplay among genetic alterations has deepened our understanding of disease heterogeneity, allowing refined risk stratification and therapeutic planning. Advances emerging from molecular research and experimental models are progressively translating into clinical practice, promoting more personalized and targeted approaches to the management of MF.

## Introduction

Myelofibrosis (MF) is a Philadelphia-negative chronic myeloproliferative neoplasm (MPN) characterized by aberrant bone marrow function and progressive fibrosis, which determine hepatosplenomegaly, extramedullary hematopoiesis, constitutional symptoms and frequently, especially in later stages, cytopenias, in particular anemia that often requires transfusion support.

Classification of MPNs, according to ICC and WHO 2022 diagnostic criteria [1, 2], includes primary MF (PMF), categorized as prefibrotic or overt fibrotic, and MF secondary (sMF) to polycythemia vera (PPV) or essential thrombocythemia (PET).

MF patients experience reduced life expectancy, with common causes of death including progression to blast phase (BP-MPN), progressive cachexia, vascular events, thrombosis and infections. To date, the only potentially curative treatment is allogeneic stem cell transplantation (ASCT), which is burdened by still relevant rates of treatment-related morbidity and mortality, and is therefore reserved for subset of eligible patients.

Constitutive activation of the JAK/STAT signaling pathway, the hallmark of MF, is driven by mutations in *JAK2*, *CALR*, and *MPL* genes, usually defined as “driver-mutations”. Alteration of this pathway leads to cytokine independence and/or hypersensitivity of mutated cells, providing a survival and proliferative advantage to mutant clones [3].

Identification of driver-mutations is crucial for MPN diagnosis, together with clinical and histopathological characteristics [1, 2]. Nevertheless, in approximately 15% of ET cases and fewer than 10% of PMF cases the driver molecular event responsible for the disease remains unidentified; those cases are defined as “triple-negative” MPN, and precise criteria are needed to reach a diagnosis in these cases, such as the presence of another clonal marker through next-generation sequencing (NGS) panels and/or cytogenetics [4].

More than 50% of MPN patients harbor at least one additional mutation in a wide range of associated-cancer genes. However, these mutations are not restricted to MPN but are shared by other myeloid malignancies including acute myeloid leukemia (AML) and myelodysplastic neoplasms (MDS). Growing evidence indicates that the number of acquired mutations increases with age, not only in patients with hematologic neoplasms but also in healthy individuals exhibiting clonal hematopoiesis of indeterminate potential (CHIP), defined as presence of myeloid disorder-associated mutation with a variant allele frequency (VAF) ≥ 2%, in the absence of severe cytopenias or a WHO-defined hematologic disorder [5].

As a result, mutational profiling has reshaped the diagnostic and classification frameworks for myeloid neoplasms, supported by increasing data demonstrating its prognostic and predictive relevance [6, 7].

Nevertheless, the relationship between genotype and phenotype in MF is more complex than in PV or ET. Whereas multiple murine models expressing *JAK2*V617F successfully reproduce ET or PV phenotypes, progression to MF appears to require additional host-specific factors or cooperating mutations that influence disease evolution and severity [8].

## DNA Methylation

### TET2

The *TET2* gene, a member of the Fe^2+^ and alpha-ketoglutarate dependent DNA-dioxygenase family, regulates hematopoietic stem cell (HSC) function through the stepwise oxidation of 5-methylcytosine to 5-hydroxymethylcytosine. Loss of function mutations -including missense, nonsense or insertion-deletion, typically heterozygous- occur in approximately 10–15% of MPN patients and represent the most common co-mutations in *JAK2*V617F-positive MPN. Data regarding the prognostic significance of *TET2* mutations remains controversial [9, 10]. *TET2* mutations enhance the in vivo repopulating ability of HSCs, driving clonal expansions even in older individuals with normal peripheral blood counts [11]. Mechanistically, *TET2* loss activates myeloid-specific transcriptional programs and pathways promoting HSCs self-renewal [12]. In xenograft models, *TET2* single-mutant clones outcompeted double-mutant clones harbouring both *TET2* and *JAK2*V617F mutations, suggesting that the addition of *JAK2* does not necessarily favours cells expansion [10]. This finding was confirmed by serial competitive transplantation experiments from Kameda et al. [13], in which *JAK2*V617F-only mutated cells showed reduced chimerism in secondary recipients and failed to promote MPN phenotype. In contrast, cooperation between *JAK2*V617F mutation and *TET2* loss supported both development and long-term maintenance of an MPN phenotype in secondary recipients, compensating for impaired HSC function, an observation further validated by in vitro sequential colony-forming assay [12].

Similar cooperative interactions were reported in MDS models, where *TET2* loss restored the impaired self-renewal capacity of *ASXL1*-deficient cells, indicating that the very particular gene may confer a selective growth advantage to HSCs [13, 14].

*TET2* depletion also disrupts normal hematopoietic differentiation, skewing toward the myeloid lineage, with resultant neutrophilia, monocytosis, and splenomegaly, features commonly observed in human CMML, suggesting that isolated *TET2* loss in the absence of other genetic lesions favours progressive myelomonocytic expansion [15].

Extensive evidence suggests that the order of mutation acquisition influences disease phenotype in MPN. Colony-forming assays have shown that patients with *TET2*-first mutations might progress to clonal hematopoiesis, whereas in *JAK2*-first patients, loss of *TET2* tends to exacerbate the clinical severity of the existing disease [16]. Another report by Ortmann et al. showed that *JAK2*-first patients were more likely to present with PV, had higher of thrombosis, and exhibited increased sensitivity to ruxolitinib in vitro, whereas *TET2*-first more commonly developed ET [17]. Notably, MF patients rarely exhibited a *TET2*-first mutation pattern, consistent with MF representing a later, more advanced disease stage [17].

### DNMT3A

The *DNMT3A* gene encodes a DNA methyltransferase essential for both de novo DNA methylation and maintenance of pre-existing methylation patterns. *DNMT3A* mutations promote HSCs self-renewal at the expense of differentiation, likely by reinforcing epigenetic programs that sustain proliferation and enable indefinite propagation of HSCs [9, 18].

These mutations are often associated with CHIP, suggesting that mutant clone progressively outcompete normal HSCs [9, 19–21]. However, single-colony sequencing and phylogenetic reconstruction of hematopoietic cells in *JAK2*-mutant MPN patients have shown that *JAK2* and *DNMT3A* mutations can occasionally arise in utero, persisting in early HSCs without advancing terminal differentiation [22]. Remarkably, *DNMT3A*-null HSCs can reconstitute haematopoiesis for at least 12 serial transplantations in mice, far exceeding the HSC normal lifespan. Molecular characterization demonstrates that this “in vivo immortalization” is accompanied by progressive and focal loss of DNA methylation at key regulatory regions controlling self-renewal genes, ultimately producing a stereotypical epigenetic and functional phenotype. Interestingly, malignant transformation in these models appears to be independent of the DNA methylation state [23].

As observed for *TET2*, the order of mutation acquisition significantly influences clinical presentation. In a study by Nangalia et al., *DNMT3A*-first mutations predisposed to ET phenotype, whereas *JAK2*V617F-first more commonly developed PV [24]. Using CRISPR/Cas9 technology, *DNMT3A* loss in a *JAK2*V617F-driven PV murine model induced progression to a fully penetrant MF phenotype, mediated by increased chromatin accessibility at enhancers regulating HSC gene expression [25]. Furthermore, Usart et al. provided strong evidence that *DNMT3A* mutations prevent *JAK2*V617F-mutant HSC from exhaustion due to interferon alfa administration, both in murine models and human cells [26].

### IDH1 E IDH2

*IDH1* and *IDH2* are key epigenetic regulators involved in both DNA methylation and histone modification and are reported in 4.5% in PMF and 1% of sMF patients. Under physiological condition, they catalyze the oxidative decarboxylation of isocitrate to a-ketoglutarate. Recurrent point mutations, most frequently R132 in *IDH1* and R140 or R172 in *IDH2*, result in accumulation of 2-hydroxyglutarate, a metabolite that inhibits multiple a-ketoglutarate-dependent enzymes, thereby leading to epigenetic dysregulation and impaired cellular responses to oxidative stress.

In MF, *IDH1/2* mutations are associated with adverse prognosis, including higher risk of transformation to BP [27]. Their prevalence is approximately 6% of MPN and up to 30% of BP [28–30].

In a large cohort of 879 PMF patients, *IDH1/2* mutations, which were mutually exclusive, occurred predominantly in association with *DNMT3A* or clustered with *SRSF2* mutations [30].

## Histone Modification

### ASXL1

The *ASXL1* (additional sex combs-like 1) gene, located on chromosome region 20q11, encodes a polycomb chromatin-binding regulator and is frequently mutated in different hematologic malignancies. *ASXL1* mutations are more common in PMF and BP (18–37%) than PV and ET (5–10%) [29–31]. In PMF, these mutations conferred an adverse impact on overall survival and increased the risk of AML transformation, even when occurring as the sole high-risk mutation [32, 33]. Furthermore, this unfavourable impact is not overcome by allogeneic hematopoietic stem cell transplantation (allo-HSCT) [34]. In contrast, sMF prognosis appears unaffected by *ASXL1* mutational status, as the presence of the mutation alone was not associated with inferior outcomes [30, 35, 36].

Clinically, *ASXL1* mutations are associated with an aggressive disease phenotype marked by leukocytosis, circulating blasts, anemia, splenomegaly and constitutional symptoms, and impact negatively on prognosis.

The underlying molecular mechanisms remain incompletely understood [30]. *ASXL1* functions as an epigenetic regulator, acting both as cofactor for the nuclear deubiquitinase BRCA1-Associated Protein 1 (BAP1) and as mediator of Polycomb Repressive Complex 2 (PRC2). Frameshift and nonsense mutations are the major types of *ASXL1* mutations, resulting in C-terminal truncation and loss of protein expression [37]. Murine models showed that both *ASXL1* loss and truncated *ASXL1* expression lead to altered hemopoiesis [38, 39]. *ASXL1* knockout in HSCs generally resulted in an increase in the total number of HSCs, increased apoptosis, altered cell cycle distribution and lead to progressive anemia and leukopenia with concomitant multilineage myeloid dysplasia [40]. Yang et al. demonstrated that transgenic expression of *ASXL1*^*aa1–587*^ truncating protein induces diverse myeloid malignancies in mice [41]. Moreover, *ASXL1* loss accelerates MF progression in *JAK2*V617F and *CALR* mutant mice [40–42]. In a *CALR*^del52^-*ASXL1*^mut^ mouse model, the phenotype shifted toward MF thorough epigenetic reprogramming, including altered H3 marks and megakaryocytes gene expression signature [43]. This event may be attributed to increased production of profibrotic cytokines which is caused by reprogramming of the fibrosis-driving potential of hematopoietic cells to fibrocytes. Specifically, *ASXL1* mutations activate the EGR1-TNFa axis, driving monocyte/macrophage inflammation and neoplastic fibrocyte-induced bone marrow fibrosis [44]. Of interest, it has been reported that *ASXL1* mutations are frequently acquired during ruxolitinib therapy as part of clonal evolution, correlating with significantly shorter overall survival after treatment discontinuation (6 vs. 16 months) [45].

In addition, a prognostic role of VAF was recently demonstrated in the GEMFIN study, where VAF > 20% predicted inferior outcomes in patients with PMF [46].

*ASXL1* mutations were also associated with a distinct methylation pattern, including both global hypermethylation [47] and lineage specific changes, suggesting a different and complex contribution to MF pathogenesis [48]. Single cell sequencing studies further demonstrated that *ASXL1*-mutant subclones exhibit unique transcriptional programs, emphasizing mutation-specific complexity at the cellular level [49].

### EZH2

*EZH2*, a histone methyl transferase member of the polycomb repressive complex 2 (PRC2), is an important regulator of chromatin topology mediating silencing through trimethylation of histone H3 on lysine 27 [50, 51].

In MPN, *EZH2* appears to act as a tumor suppressor; different loss-of-function mutations have been identified, often cooperating with *JAK2*V617F to promote MF. In murine models, the expression of *JAK2*V617F, in the background of *EZH2* knockout mice, induced skewing toward megakaryopoiesis, with marked thrombocytosis or leukocytosis, progression to MF and reduced survival, compared to the *JAK2*-mutated only mice. As previously reported, members of TNF/NF-kB were enriched in these mouse models [52–54]. These observation mirror findings in patients where *EZH2* mutations correlate with a higher leukocyte count, blast count, and larger spleen at diagnosis, serving as an independent poor prognostic factor for reduced overall survival, as part of the high-molecular-risk (HMR) group of mutations [30, 55].

## m-RNA Splicing

Mutations in RNA splicing machinery components (*SF3B1*, *SRSF2*, *U2AF1*, *ZRSR2*, and others) are recurrent across myeloid malignancies, particularly MPN or MDS. These mutations are typically mutually exclusive and heterozygous missense, with dominant negative activity that results in disruption of RNA splicing [56].

*SRSF2* gene is the most frequently mutated in MPN, with hotspots at proline 95 residue. As a member of the serine/arginine-rich protein family, it regulates both constitutive and alternative splicing by binding exonic splicing enhancer (ESE) sequences within pre-mRNA [57]. Mutations alter RNA binding specificity, leading to mis-splicing of key hematopoietic regulators [58].

Clinically, *SRSF2* mutations occur in 8–22% of PMF and BP, while being less frequent in PV and ET patients, and are associated with poor overall and leukemia-free survival [59–62]. Although they often cluster with *IDH* mutations, they retain negative independent prognostic value [59]. The poor prognosis translates into an aggressive clinical phenotype, such as older age, leukocytosis, circulating blasts and constitutional symptoms [30]. Mutated *SRSF2* is one of the HMR genes in MF.

Interestingly, while *SRSF2* mutations increase the risk of leukemic transformation, they do not seem to promote progression from prefibrotic to fibrotic MF, in contrast to *ASXL1* or *EZH2*. In murine transplantation experiment, *SRSF2*P95H unexpectedly attenuated *JAK2*V617F-induced MF phenotype and decreased TGFβ1 serum levels, possibly by promoting aberrant *JAK2* exon 14 skipping, which produces an inactive protein and downregulates JAK-STAT signalling [63]. Moreover, co-expression of *SRSF2*P95H reduced the competitiveness of *JAK2*V617F mutant HSCs [64].

*U2AF1* mutations occur mainly at the hotspot residues Q157 and S34, and are present in 5–15% of PMF and BP. They are associated with shorter overall survival [56, 65], meriting its inclusion in the MIPSS70-plus v2.0 and GIPSS [33, 66], MIPSS70 + v2.0 prognostic model [32, 33]. Clinically, *U2AF1* mutations associate with anemia or thrombocytopenia, likely due to impaired erythroid differentiation and lineage-specific splicing defects [67]. Experimental evidence suggest that *U2AF1* mutation enhance megakaryocytic differentiation in vivo, impair mitochondrial function and disrupt DNA damage response [68].

*SF3B1* mutations, a component of the U2-small nuclear ribonucleoprotein complex, are uncommon in MF and are reported in < 10% of cases [69], being more frequent in MDS and MDS/MPN, where they represent a disease-defining genetic event. In PMF they generally lack prognostic impact [70], although in sMF they were. reported to be associated with reduced survival [71].

Finally, *ZRSR2* mutations are very rare in MPN, more commonly found in PMF. They can occur through the entire gene and are generally loss of function. No clear prognosis impact has been identified. In particular, the aberrant splicing seems to be insufficient to drive fibrotic progression of MPN [72]. As in MDS, they cluster with *TET2* mutations [73].

## RAS-Pathway

The RAS/MAPK pathway regulates multiple cellular functions through kinase-mediate signaling [74].

*CBL* mutations affect a multifunctional adaptor protein with ubiquitin ligase activity, leading to stabilization of receptor tyrosine kinases, resulting in constitutive activation of signalling pathways, cytokine hypersensitivity, and autonomous cell proliferation. They can be detected in up to 6% of PMF and BP patients and are mostly missense [75].

Missense mutations in *NRAS/KRAS*, especially in codons 12, 13, and 61, drive constitutive activation of growth signalling [76]. Distinct *RAS* mutations display variable effects in murine models. The induction of heterozygous *KRAS*^G12D/+^ expression in the hematopoietic system alone leads to a rapid and highly penetrant myeloproliferative phenotype [77], while heterozygous NRAS^G12D^ produces a milder phenotype [78]. Furthermore, in murine models the cooperation of RAS mutations with other genes, such as *TET2*, *DNMT3A*, or *TP53*, determined an aggressive phenotype that frequently evolve to acute leukemia [79].

Clinically, *CBL/NRAS/KRAS* mutations in PMF patients were associated with adverse clinical features, shorter overall survival, and poor response to JAK inhibitor therapy [80], although there are not included in the MIPSS70/2.0 scores because was not observed a risk redistribution when considering those mutations, suggesting that such mutations do not add relevant practical information [81].

## Transcription Factors

Among transcription factors, *RUNX1* and *NFE2* are most frequently mutated in MPNs.

*NFE2* gene regulates megakaryopoiesis and erythropoiesis. Mutations include frameshift variants and deletions [82, 83]. Elevated *NFE2* levels in vivo were shown to induce an MPN phenotype and predispose to leukemic transformation in transgenic mice; in particular truncated *NFE2* proteins enhance wild-type *NFE2* function and cause erythrocytosis and thrombocytosis, imparting a proliferative advantage [84]. Mutant *NFE2* mice are prone to acquire additional genetic lesions, promoting leukemogenesis [85].

*RUNX1* mutations are enriched in BP and are associated with shorter overall survival [6]. Mice lacking both *RUNX1* and *RUNX2* in mesenchymal stem cells display an increase in fibrosis and bone formation with markedly reduced HSCs in bone marrow [86].

### TP53

*TP53* gene plays a critical role in cellular repair and apoptosis. *TP53* mutations are among the strongest predictors of poor prognosis in hematologic malignancies, including MPNs. In particular, the role of single-hit *TP53* mutation is still debated, since mutations can be detected in a heterozygous state at a low allelic burden for an extended period of time without clonal expansion. By contrast, increasing evidence supports the unfavourable impact of multi-hit *TP53*. Numerous studies have confirmed that *TP53* loss promotes leukemic transformation in murine models [87]. Data from retrospective studies showed that overall survival in chronic phase MF was significantly shorter with multi-hit versus non-multi-hit *TP53* mutations, independent of other risk factors [88]. Gagelmann et al. further demonstrated that allelic configuration or karyotypic abnormalities influence post-ASCT outcomes in MF patients [89].

*TP53* mutations, particularly multi-hit, although not integrated in standard risk scores, should be taken into account when evaluating patients, owing to their independent role in predicting a dismal outcome [81].

## Other genes

Somatic *SETBP1* mutations are found in various myeloid disorders covering both MPNs and MDS [90]. The mutations target the substrate recognition domain of the E3 ubiquitin ligase, affecting ubiquitin binding, and are usually a late clonal event. In a mouse model expressing *SETBP1*^G870S^, mice developed a chronic myeloid disorder resembling MF, caused by an extensive alteration of the normal hematopoietic differentiation program. Intriguingly, some patients diagnosed with triple negative PMF were found to carry *SETBP1* mutations as the first hit, suggesting that the timing of appearance of *SETBP1*mutations along tumor history influences the disease phenotype [91].

*PTPN11* mutations are overall rare in MPNs and mainly in BP, where are linked to poor overall survival [20]. *PPM1D* mutations lack a clear correlation with prognosis but resulted to be related with treatment resistance on other myeloid malignancies and were recently described in 1.9% of patients with MPN [6].

*LNK-SH2B3* is an adaptor protein that physiologically binds *JAK2* and negatively regulates JAK-STAT signaling [92]. Missense substitutions can be found both in idiopathic erythrocytosis as a germline variant and acquired mutation in MPN, either alone or in association with a driver mutation. Some of *SH2B3* mutations are predicted to be hypomorphic mutations (resulting in a reduced level of activity) by structural analysis of their location within the functional domains, whereas others are predicted to be damaging or deleterious with three different prediction software [93, 94]. In mouse models, LNK loss exacerbates *JAK2*V617F phenotype with promotes rapid development of MF [95] and was associated in univariate analysis with shorter leukemia-free survival, but not confirmed in multivariable analysis [9]. Finally, a large study based on sequencing of 69 genes in more than 2000 patients with MPN identified novel mutations in *MLL3* and *GNAS* genes, and there’s also increasing evidence that mutations in genes typically involved in other myeloid neoplasms such as *DDX41* [96] or *ETV6* [97] may have an impact in MPNs. Details about mouse models in MF are described in Table 1.

**Table 1:** Mouse models of myelofibrosis

Gene	Model	Type	Strain	Locus	Phenotype	References
*TET2*						
	*TET2* ^fl/fl^ Mx1-Cre	Knock-out (conditional)	C57BL/6	Endogenous	Increased HSC self-renewal, myeloid expansion, favors MPN/MDS-like transformation	[12]
	*TET2* ^fl/fl^ Vav-Cre	Knock-out (hematopoietic)	C57BL/6	Endogenous	Increased HSC self-renewal, myeloid skewing	[12]
	Human CD34⁺ xenotransplant	Humanized (xenograft)	NOD-SCID	/	Preserved multilineage colony-forming capacity	[10]
	*TET2*−/−	Knock-out (constitutive)	C57BL/6	Endogenous *TET2* locus disrupted by nlacZ/nGFP cassette	Expanded HSC pool and self-renewal, present with monocytosis, splenomegaly; CMML-like or MDS/MPN-like	[98]
	*TET2*−/− *JAK2*^V617F^	Knock-out (*TET*2) Knock-in (*JAK2)*	C57BL/6	Endogenous	Accelerated MPN progression, increased myeloid expansion	[13]
	*TET2*−/− *JAK2*^V617F^	Knock-out (*TET2*) Knock-in (*JAK2*)	C57BL/6	Endogenous	Enhanced HSC self-renewal, increased fibrosis; mutation order influences phenotype	[14]
*DNTM3A*						
	*DNMT3A* ^fl/fl^	Knock-out (conditional)	C57BL/6	/	Impaired HSC differentiation, self-renewal bias rather than differentiation, reduced DNA methylation	[18]
	*DNMT3A-/-* *JAK2* ^V617F^	Knock-out (*DNMT3A*) Knock-in *(JAK2*)	C57Bl/6	/	PV with progression to MF in combined model	[25]
*ASXL1*						
	*ASXL1*^*MT*^	Knock-in (truncation)	C57BL/6	Rosa26	Maintained survival in competitive transplantation; increased HSC susceptibility to leukemic transformation	[38]
	*ASXL1* ^f/f^	Knock-out	C57BL/6	/	Defective erythroid progenitors; dysregulated erythropoiesis	[39]
	*ASXL1* ^*Y588X*^	Transgenic (gain of function)	C57BL/6		Enlarged HSC pool; shortened survival; broad myeloid malignancy predisposition	[41]
	*ASXL1−/− JAK2* ^*V617F*^	Knock-out (*ASXL1*) Knock-in (*JAK2*)	C57BL/6	Endogenous	Accelerated MF progression, increased megakaryopoiesis, cytokines, HSC exhaustion	[42]
	*ASXL1* ^MT^ *CALR* ^del52^	Knock-in (*ASXL1*, truncation) Knock-in (*CALR*^del52^)	C57BL/6	Rosa26 Endogenous *CALR*	Aggressive MPN, higher platelet counts, early-onset fibrosis, altered H3K27me3/H3K4me3 in megakaryocyte	[43]
	*ASXL1* ^MT^ *ASXL1−/−*	Knock-out or truncating gain of function	C57BL/6	Endogenous	Accelerated fibrosis, expansion of neoplastic fibrocytes expansion via EGR1–TNFα axis	[44]
*EZH2*						
	*EZH2* ^fl/fl^ *JAK2*^V617F^	Knock-out (*EZH2*) Knock-in (*JAK2*)	C57BL/6	Endogenous	Rapid onset of MPN and fibrosis progression, marked splenomegaly, increased inflammatory cytokines	[52]
	*EZH2* ^fl/fl^ *JAK2*^V617F^	Knock-out (*EZH2*) Knock-in (*JAK2*)	C57BL/6	Endogenous	Severe MF, increased TGF-β, pro-fibrotic pathways, MK expansion; reduced H3K27me3	[53]
*SRSF2*						
	*SRSF2* ^P95H^	Knock-in (conditional)	C57BL/6	Endogenous	Reduced JAK/STAT signaling, pSTAT5 and myeloid expansion; delayed MF progression	[63]
	*SRSF2* ^P95H^ *JAK2*^V617F^	Knock-in (*SRSF2*) Knock-in (*JAK2*)	C57BL/6	Endogenous	Reduced erytrhopoiesis, progenitors function, fibrosis; attenuated MPN phenotype	[64]
*RAS*						
	*KRAS* ^G12D^	Knock-in (conditional LSL-G12D)	C57BL/6	Endogenous	Aggressive MPN phenotype, granulocyte/monocyte expansion, splenomegaly, HSC hyperproliferation with exhaustion	[77]
	*NRAS* ^G12D^	Knock-in (conditional)	C57BL/6	Endogenous	MPN/CMML-like disease, monocytosis, splenomegaly, AML progression	[77]
*OTHER*						
*TP53*	*TP53*−/− *JAK2*^V617F^	Knock-out (*TP53*) Knock-in (*JAK2*)	C57BL/6	Endogenous	Progression to AML, HSC dysregulation	[87]
*SETPB1*	*SETPB1* ^D868N^ *SETPB1*^I871T^	Knock-in (gain-of-function, stabilizing)	C57BL/6	Endogenous	Aggressive MPN, leukocytosis, early progression to MF, increased HSC expansion, HOXA9/10 upregulation	[91]
*LNK*	*LNK*−/−	Knock-out	C57BL/6	Endogenous	HSC and myeloid expansion, loss of quiescence, increased JAK2 signaling	[92]
*LNK*	*LNK−/−*	Knock-out	C57BL/6	Endogenous	Leukocytosis, megakariocytic expansion, splenomegaly	[94]

## Conclusions

The rapid accumulation of mutation profiling data in clinical practice has profoundly transformed our understanding of MF biology. Recent advances in single-cell sequencing technology have enabled a more accurate exploration of the clonal architecture and dynamics of neoplastic cell evolution, providing new insights into the mechanisms underlying disease initiation and progression. The genetic landscape of MF extends far beyond the canonical driver mutations in *JAK2*, *CALR*, and *MPL.* A much broader landscape of non-driver somatic mutations contributes to disease biology, influencing clinical phenotype, risk of progression and overall survival. Among these, *ASXL1*,* EZH2*,* SRSF2* and *IDH1/2* (together with *U2AF1* included in MIPSS70 plus 2.0 model) are classified as high-molecular-risk mutations due to their strong prognostic impact. However, it is now evident that in addition to HMR, the number itself of additional somatic mutations affect prognosis, and other mutations, particularly *TP53*, although not yet formally integrated into prognostic models, exert a major influence on clinical outcomes, predicting poor survival and response to treatment. These evidences underscore the need for their systematic evaluation in clinical decision-making. Incorporating molecular data into clinical practice is essential for risk stratification, prognostication and treatment decision-making. Ultimately, the complexity of myeloid neoplasms arises from the interplay between gene function, mutation type, order of acquisition, functional consequences at cellular level, and interactions with other genes or/and bone marrow microenvironment. Cooperation among multiple genetic and epigenetic lesions drives disease heterogeneity, progression, and therapeutic resistance. Deciphering these mechanisms is essential to refine prognostic models, guide personalized therapy, and improve long-term outcomes. A deeper understanding of these pathways may pave the way to improve long term outcomes and survival by developing targeted therapies -such as IDH inhibitors, splicing modulators or epigenetic drugs-, hopefully expanding therapeutic options for MF in the near future.

## Data Availability

No datasets were generated or analysed during the current study.
